# ﻿*Hemiboeakaiyangensis*, a new species of Gesneriaceae endemic to Guizhou, China

**DOI:** 10.3897/phytokeys.211.85630

**Published:** 2022-10-14

**Authors:** Tao Peng, Shun-Zhi He, Shun-Li Wang, Dan Huang, Xu-Ping Zhou

**Affiliations:** 1 Biodiversity Research Center, School of Life Sciences, Guizhou Normal University, CN-550025 Guiyang, China Guizhou Normal University Guiyang China; 2 Guizhou University of Traditional Chinese Medicine, CN-550025 Guiyang, China Guizhou University of Traditional Chinese Medicine Guiyang China

**Keywords:** Gesneriad, ITS, morphology, phylogeny, taxonomy, *trn*L-F

## Abstract

A new species of Gesneriaceae from Guizhou, China, *Hemiboeakaiyangensis***sp. nov.**, is described and illustrated. We investigated its phylogenetic position and relationships with 13 other species of *Hemiboea* C.B.Clarke, which present large morphological diversity in the genus, based on molecular analyses of the nuclear ribosomal internal transcribed spacer (ITS) and the chloroplast *trn*L-F intron-spacer sequences. The molecular phylogenetic analyses revealed that the new species is most closely related to *H.ovalifolia*. A diagnostic table and discussion of morphological characters are provided to differentiate the new species from *H.longisepala*, *H.flaccida* and *H.ovalifolia*.

## ﻿Introduction

The genus *Hemiboea* C.B.Clarke has traditionally been divided into two sections, sect. Subcapitatae C.B.Clarke and sect. Hemiboea (Li 1987a, 1987b). Many species of this genus can be found on limestone hills in tropical and subtropical evergreen broadleaved forests. Most species of *Hemiboea* are located in southern and southwestern China, while only a few are located outside China. For instance, *H.subcapitata* C.B.Clarke and H.cavalerieiLévl.var.paucinervis W.T.Wang & Z.Y.Li ex Z.Y.Li are also found in northern Vietnam; *H.bicornuta* (Hayata) Ohwi is distributed in Taiwan Island of China and the Ryukyu Islands of Japan (Li and Wang 2005).

So far, a total of 38 species and six varieties have been described in *Hemiboea* (Li and Liu 2004; Xu et al. 2010; Huang et al. 2011; Wen et al. 2011; Pan et al. 2012; Xu et al. 2012; Wen et al. 2013; Zhou et al. 2013; Zhang et al. 2014; Huang et al. 2017; Chen et al. 2018; Li et al. 2018; Wei 2018; Li et al. 2019; Nguyen et al. 2019; Wu et al. 2019; Huang et al. 2020; Nguyen et al. 2021). The small genus *Metabriggsia* W.T.Wang was established based on two species, *M.ovalifolia* W.T.Wang and *M.purpureotincta* W.T.Wang from Guangxi, China (Wang 1983). Some similarities in corolla morphology with certain species of the former *Briggsia* Craib are reflected in the generic name (Wang 1983), though *Metabriggsia* is morphologically similar to *Hemiboea*, and in recent molecular-based taxonomic studies, the two species of *Metabriggsia* were sunk into *Hemiboea* as *H.ovalifolia* (W.T.Wang) A.Weber & Mich.Möller and *H.purpureotincta* (W.T.Wang) A.Weber & Mich.Möller (Weber et al. 2011).

During the course of a 2009 floristic study in Guizhou Province, China, we collected specimens of an unidentified *Hemiboea* from a limestone area in Kaiyang County. It seemed a species of the sect. Subcapitatae of the genus *Hemiboea* because of the free calyx lobes and elongate corolla. However, it differed from all known species in this genus. We also observed that its vegetative morphology was similar to that of *H.ovalifolia* because of the stems covered with long white pubescent hairs, and the leaf blades herbaceous and ovate or ovate-oblong. The inflorescence and flower morphology indicated that this species belongs to *Hemiboea* as previously circumscribed; for example, it possesses involucre, its corolla is infundibular-tubular, and has a ring of hairs on the interior above the corolla base. After consulting national floras and the relevant literature (Li 1983, 1987a, b; Wei and Wen 1995; Weitzman et al. 1997; Wang et al. 1998; Li and Liu 2004; Li and Wang 2005; Wei et al. 2010; Xu et al. 2010), as well as herbarium specimens, we confirmed that it is an undescribed species. We described and illustrated the new species herein. We used molecular data of the nuclear ribosomal internal transcribed spacers (ITS) and the chloroplast *trn*L-F intron-spacer (*trn*L-F) to confirm the placement of the newly collected material in *Hemiboea* and to infer its phylogenetic relationships with other species in the genus.

## ﻿Methods

### ﻿Taxon sampling

To investigate the phylogenetic placement of the newly collected taxon, our sampling focused on species of *Hemiboea* and its closest related genera according to Möller et al. (2011) and Weber et al. (2011). The ITS and *trn*L-F sequences used in Weber et al. (2011) included 13 species of *Hemiboea* and four outgroup samples (one species of *Ornithoboea*, one of *Paraboea* and two of *Glabrella*) were downloaded from Genbank (Table 1). Leaf material of the new species was collected from living plants in the type locality and rapidly dried in silica gel.

**Table 1.** Species names and accession numbers of ITS and *trn*L-F sequences used for phylogenetic analysis.

Species	Voucher number	trnL-F	ITS
**Outroup samples**			
*Briggsialongipes* (Hemsl. ex Oliv.) Craib	MMO 01-122	FJ501545	AF055052/AF055053
*Briggsiamihieri* (Franch.) Craib	Y.Z.Wang 11315B	FJ501544	FJ501363
*Ornithoboeawildeana* Craib	Middleton & al. 4531	JN934710	JN934752
*Paraboearufescens* (Franchet) B.L.Burtt	Möller MMO 01-108/3	JN934730	JN934772
**Ingroup samples**			
*Hemiboeaovalifolia* W.T.Wang (W.T.Wang) A.Weber & Mich.Möller	B.M.Nong 06-1	HQ632883	HQ632980
*Hemiboeapurpureotincta* (W.T.Wang) A.Weber & Mich.Möller	MMO 06-813	HQ632884	HQ632981
*Hemiboeabicornuta* (Hayata) Ohwi	RBGE cult. 19951207	FJ501534	FJ501356
*Hemiboeacavaleriei* Lévl.	Z.J.Gu G3	FJ501533	FJ501355
*Hemiboeafangii* Chun ex Z.Yu Li	MMO 08-1284	HQ632882	HQ632979
*Hemiboeafollicularis* C.B.Clarke	Y.G.Wei G03	HQ632885	HQ632982
*Hemiboeagracilis* Franchet	Y.Z.Wang 11317	FJ501536	*
*Hemiboealonggangensis* Z.Yu Li	Y.G.Wei 07-550	HQ632889	HQ632986
*Hemiboealongzhouensis* W.T.Wang	MMO 07-1127	HQ632888	HQ632985
*Hemiboeaomeiense* W.T. Wang	MMO 08-1271	HQ632886	HQ632983
*Hemiboeakaiyangensis* T. Peng & S.Z.He, sp. nov.	Shun-Zhi He, 090819	JN644339	JN644335
*Hemiboearubribracteata* Z.Yu Li & Yan Liu	MMO 07-1093	HQ632890	HQ632987
*Hemiboeasubcapitata* C.B.Clarke	Y.Z.Wang 11306	FJ501535	FJ501357

* – Wei et al. 2010*Litostigma* paper.

### ﻿DNA extraction, PCR and direct sequencing

Molecular methods and protocols followed Möller et al. (2009) and Weber et al. (2011). The Genbank accession numbers for ITS and *trn*L-F of the new species are JN644339 and JN644335, respectively (Table 1).

### ﻿Phylogenetic analysis

Sequences of ITS and *trn*L-F of the new species were aligned with the existing matrices of Weber et al. (2011), and the combined data analyzed by Möller et al. (2009, 2011) and Weber et al. (2011). All characters were unordered and equally weighted. Heuristic searches were implemented as 1000 random taxon-addition sequences, with tree bisection–reconnection (TBR) branch swapping, the MULTRESS and STEEPEST DESCENT option in effect. Branch support was obtained by bootstrapping (Felsenstein, 1985ab) with 1000 random resamplings and TBR on and MULTREES off (Möller et al. 2009).

## ﻿Results

The combined dataset included 1490 characters and contained 1125 (75.5%) constant sites, 333 (22.3%) variable sites and 154 (10.3%) parsimony-informative sites. The heuristic analysis resulted in three most parsimonious trees with a length of 553 steps, a consistency index (CI) of 0.7776, and a retention index (RI) of 0.6295. The strict consensus tree supports that the new species nests in a strongly supported clade of *Hemiboea* (BS = 100%), and it is the sister taxon to *H.ovalifolia* (BS = 99%) (Fig. 3).

## ﻿Discussion

The molecular phylogenetic analysis revealed that the new collection *Hemiboeakaiyangensis* fell into the clade of *Hemiboea*, and its most closely related species was *H.ovalifolia*, which is congruent to the morphological evidence. Morphologically, this species is most similar to *H.longisepala*, *H.flaccida*, and *H.ovalifolia*, and it can be easily distinguished by the characters summarized in Table 2.

**Table 2.** Diagnostic characters used to differentiate *Hemiboeakaiyangensis* from most similar taxa.

Taxon		*Hemiboeakaiyangensis*	*H.longisepala*	*H.flaccida*	*H.ovalifolia*
**Stem**		densely pubescent	Glabrous	densely brown puberulent to villous	brown villous
**Leaf blade**	**Texture**	herbaceous	Papery	slightly fleshy	herbaceous;
	**Shape**	ovate or ovate-oblong	ovate-lanceolate to elliptic-lanceolate	elliptic to ovate	ovate
	**Hairs**	appressed pubescent on adaxially and abaxially surface	adaxially sparsely pubescent, abaxially glabrous	pubescent to densely on adaxially and abaxially surface	appressed pubescent on adaxially and abaxially surface
	**Lateral veins**	7–10 on each side	10–12 on each side	5–8 on each side	5–10 on each side
**Cyme**	**Peduncle(cm)**	10–18	3–3.6	0.4–1.9	7.5–12.5
	**Hairs on peduncle**	densely white long glandular pubescent	glabrous	sparsely glandular puberulent to pilose	brown glandular pubescent
	**Involucre**	cordate, early deciduous	spheroidal, ca. 1.7 cm in diam.	nearly spheroidal, 1–2.5 cm in diam., outside sparsely glandular puberulent	nearly spheroidal, early deciduous
	**Calyx lobes**	oblong-lanceolate, apex obtuse or slightly obtuse, 12–13 × 2.5–3 mm, outside densely glandular pubescent, glabrous inner, 3 veins on one lobe	linear-lanceolate, 19–20× ca. 2.5 mm, outside and margin glabrous	linear, 5–9 × 2.5–3 mm, outside and margin sparsely glandular puberulent	lanceolate-linear, 9–10 × 1.5–2 mm, pubescent outer, glabrous inner, 3–5 veins on one lobe
**Flower**	**Colour**	outside pale yellowish-green to pale yellowish-white	outside white	outside white, inside purple spotted	white, suffused yellow-green
	**Hairs**	outside densely glandular puberulent, inside glabrous	outside glabrous	outside sparsely glandular puberulent, inside glabrous	outside sparsely pubescent near apex, inside glabrous
	**Size**	4.5–5 cm long; tube 3.5–4 cm; adaxial lip 6–7 mm, abaxial lip 6–7 mm	ca. 3.4 cm long, tube ca. 2.6 cm; adaxial lip ca. 6 mm; abaxial lip ca. 8 mm	3–3.4 cm long, tube 2.3–2.5 cm; adaxial lip 4–5 mm; abaxial lip 7–9 mm	ca. 3.6 cm long, tube ca. 2.7 cm; adaxial lip ca. 2.8 mm; abaxial lip ca. 1 cm
	**Staminodes**	2, ca. 7 mm long	3, central 1 ca 1.5 mm long, lateral 5.5 mm	2, 6–8 mm long	3, central 1 ca. 1.5 mm long, lateral 2, 9–10 mm
	**Pistil**	glabrous	glabrous	sparsely glandular puberulent	sparsely puberulent

## ﻿Taxonomic treatment

### ﻿ Hemiboea kaiyangensis

Taxon classificationPlantaeLamialesGesneriaceae

T.Peng & S.Z.He sp. nov.

484E8086-12B5-5D54-9D84-71365E7B7B3C

urn:lsid:ipni.org:names:77306628-1

Figs 1 , 2 , 3

#### Type.

**Figure 1.**
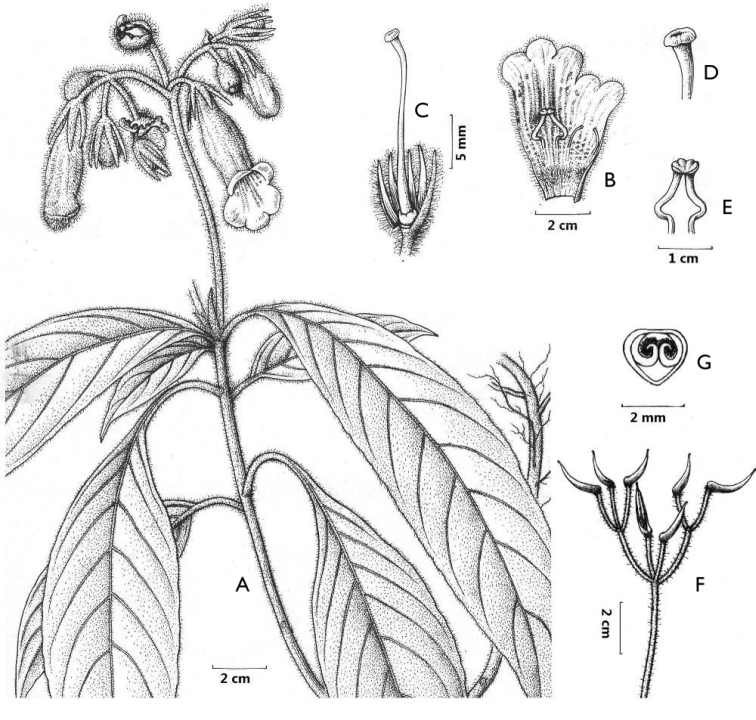
*Hemiboeakaiyangensis* T.Peng & S.Z.He **A** habit, showing flowering branch **B** opened corolla, showing stamens, staminodes and ring of hairs at base of tube **C** calyx, pistil and disc **D** stigma **E** stamens **F** infructescence **G** cross section of ovary, showing parietal placentation. Drawing by S.Q.He and Y.X.Zhu.

**Figure 2.**
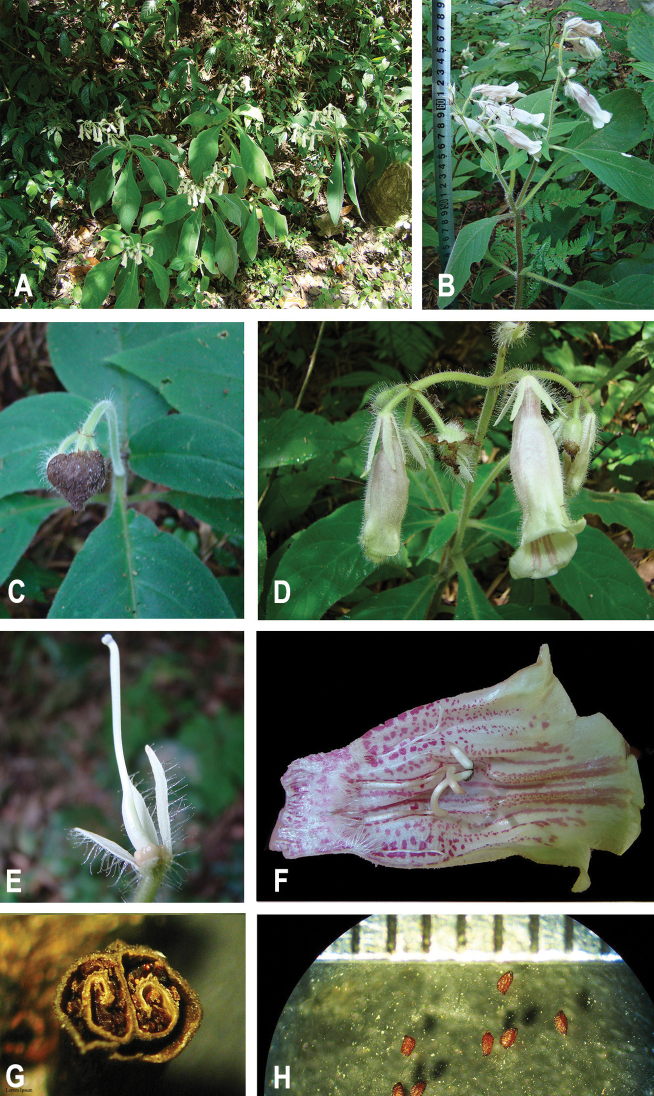
*Hemiboeakaiyangensis* T.Peng & S.Z.He **A** habitat **B** plant with flowering branches **C** involucrum, early deciduous, before flower opening **D** pair-flowered cymes **E** calyx, disc and pistil **F** opened corolla, showing stamens, staminodes and ring of hairs at base of tube **G** cross section of capsule, showing two parietal placentation **H** seeds. Scale bar: 0.5 mm (Based on the holotype *Shun-Zhi He 90819*).

China; center of Guizhou Province, Zijiang gorge, Kaiyang County, grows on cliffs under forests along the road; alt. 1000–1020 m. 2009-08-18, *Shun-Zhi He* 90819 (Holotype: HGCM!, isotype: GNUB!, IBK!) (Figs 1, 2).

#### Diagnosis.

*Hemiboeakaiyangensis* is most similar to *H.longisepala* Z.Y.Li, *H.flaccida* Chun ex Z.Y.Li and *H.ovalifolia* (W.T.Wang) A.Weber & Mich.Möller in the glabrous pistil, but it differs in the cordate involucre bracts that are early deciduous, the corolla densely glandular puberulent outside and glabrous inside, and pale yellowish-green to pale yellowish-white outside.

#### Description.

Perennial herb. Rhizomatous. Stems 25–60 cm long, 5–7 mm in diam., densely pubescent. Petiole 0.5–4.5 cm long, densely pubescent. Leaf blade herbaceous, oblique, iso- to distinctly anisophyllous, ovate or ovate-oblong, 13–26 × 5–8 cm, apex acute, base oblique cuneate, margin nearly entire or unapparent sinuous dentate, appressed pubescent on both sides, lateral veins 7–10 on each side. Cymes 2–3, terminal or subterminal, 6–12 flowers per cyme; peduncle 10–18 cm long, densely pubescent with white long glandular hairs; involucre cordate, apex cuspidate, early deciduous; pedicel 0.7–1.5 cm long, pubescent with white long glandular hairs. Calyx lobes 5; lobes oblong-lanceolate, apex obtuse or slightly obtuse, 12–13 × 2.5–3 mm, outside densely pubescent with glandular hairs of 3–4 mm long, inside glabrous, 3 veins per lobe. Corolla pale yellowish-green to pale yellowish-white outside, small purplish-brown spotted inside, 4.5–5 cm long, densely glandular-puberulent outside, glabrous with a ring of white hairs ca. 4 mm above the corolla base inside; tube 3.5–4 cm long, mouth 1–1.3 cm in diam.; limb distinctly 2-lipped, adaxial lip 2-lobed, lobes obliquely semicircular, apex obtuse to rounded, 6–7 mm long, 8–9 mm in diam. at the base of lobes; abaxial lip tripartite, lobes margin ciliolate, the central broadly ovate to ovate-elliptic, 6–7 mm long, two lateral oblique triangle, 7–8 cm long. Stamens 2, glabrous, adnate to 1.8 cm above the corolla base, filament ca. 1.3 cm long, geniculate at the middle; anthers slightly oblong, dark purple, apex coherent. Staminodes 2, glabrous, adnate to 1.7 cm above the corolla base, ca. 7 mm long. Nectary disc ring-like, 1.1–1.2 mm high, atop with ca. 6 obviously erose crena. Pistil 2–2.8 cm long, ovary 7–9 mm long, glabrous, style 1.4–2 cm long, glabrous, stigma 1, terminal truncate, sightly 2-lobed. Capsule obliquely linear-lanceolate, 2–2.4 cm long, 3–3.3 mm in diam., glabrous, slightly curved.

**Figure 3.**
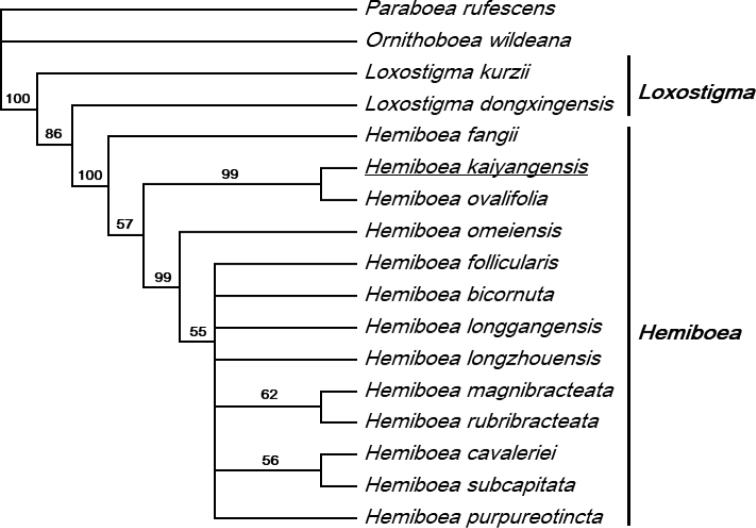
Strict consensus tree of three parsimony trees based on combined ITS and *trn*L-F data. Numbers above branches are bootstrap values. Underline indicates the new species.

#### Pollen description.

Pollen grains of *Hemiboeakaiyangensis* are prolate-spheroidal, long or oblate, 3-colporoidate grains. In polar view, the outline is close to triangular-circular. The ectocolpi measures 33.05–33.57 × 12.15–14.23 μm and the endoapertures are laterally fused to form an endocingulum. Exine reticulate, muri smooth. The width of muri is unequal in size. The sizes and shapes of perforations are irregular, and vary in size from 0.14–0.67 × 0.11–0.61 μm.

#### Distribution and ecology.

Known only from a single limestone gorge in Kaiyang county, Guizhou Province, China. Only five populations were found, growing on the mouth of caves in shady and damp forests, close to a road, between 900 and 930 m in elevation.

#### Etymology.

The name of the new species, *kaiyangensis*, refers to the type locality, Kaiyang County, Guiyang, Guizhou Province, China.

#### Conservation status.

The populations of *Hemiboeakaiyangensis* are endemic to Kaiyang county, center of Guizhou Province, China, and the species only known from the type locality at present. The five detected populations grow dispersed in a limestone gorge, and cover only an area of about 1.25 km^2^ and include a total of 75–120 individuals. However, until further investigation, the species should be designated as “Data Deficient” (DD) according to the IUCN standards (IUCN 2019).

#### Notes.

As previously mentioned, *Hemiboeakaiyangensis* is most morphologically similar to *H.longisepala*, *H.flaccida* and *H.ovalifolia* in their glabrous pistil, but some characters, such as the early deciduous and cordate involucre bracts, the corolla indumentum (outside densely glandular puberulent and inside glabrous), and the corolla color (outside pale yellowish-green to pale yellowish-white), help us to easily distinguish them. Specifically, *H.kaiyangensis* is similar to *H.flaccida* in having two staminodes, but it differs in the longer peduncle (10–18 cm), the involucre cordate but early deciduous, the bigger calyx lobes, oblong-lanceolate (12–13 × 2.5–3 mm), and the glabrous pistil. This new species is also similar to *H.ovalifolia* in the texture and shape of leaf blade, but it can be distinguished in the peduncle indument, with dense white long glandular hairs, the involucre cordate, the calyx lobes outside densely glandular pubescent, the corolla outside densely glandular puberulent, 2 staminodes, and the pistil glabrous. Lastly, *H.kaiyangensis* is similar to *H.longisepala*, but it differ from the latter in the stem densely pubescent, the longer peduncle (10–18 cm), the peduncle with dense white long glandular hairs, the corolla outside densely glandular puberulent and 2 staminodes. All compared details of four congeners were listed in Table 2.

## Supplementary Material

XML Treatment for Hemiboea kaiyangensis
